# Effect of Fe Concentration and Superheating on the Microstructure and Tensile Properties of High Mg 413.0-Type Alloys: Role of Sr, Be, P, and La

**DOI:** 10.3390/ma18020249

**Published:** 2025-01-08

**Authors:** Herbert W. Doty, Ehab Samuel, Agnes M. Samuel, Ehab Elsharkawi, Victor Songmene, Fawzy H. Samuel

**Affiliations:** 1 Materials Technology, General Motors Global Technology Center, Warren, MI 48092, USA; herb.doty@gm.com; 2 Département des Sciences Appliquées, Université du Québec à Chicoutimi, Saguenay, QC G7H 2B1, Canada; ehabfhsamuel@gmail.com (E.S.); agnesmsamuel@gmail.com (A.M.S.); 3 Division of Engineering, Saint Mary’s University, Halifax, NS B3H 3C3, Canada; ehab.elsharkawi@smu.ca; 4 Department of Mechanical Engineering, École de Technologie Supérieure, Montreal, QC H3C 1K3, Canada; victor.songmene@etsmtl.ca

**Keywords:** aluminum alloys, superheating, Fe content, Sr modification, Al_2_Si_2_Sr phase

## Abstract

The present work was undertaken to explore the multiple alloys and process steps that have been suggested to mitigate the harmful effects of high iron content in cast Al-Si alloys. The base alloy used was ommercial 413.0 alloy containing 0.35%Mg. Iron was added at three Fe levels up to 1.8%. The addition of Sr, 1.0%Zn, 0.2%Ti were made to the alloys so prepared, which were melted and maintained at a superheat of 750 °C or 950 °C. The melts were poured in different molds that produced three solidification rates. In total, 40 castings were prepared: half of the castings were used for metallographic examinations in the as-cast condition, while the other half were set aside for tensile testing following T6 treatment. The results show that at a solidification rate of 50 °C/s, 1.8%Fe could be dissolved in the aluminum matrix regardless of other melt treatments. With regard to the other solidification rates, superheating at 950 °C, coupled with Sr addition or Sr + Be, reduces the average β-platelets length by 80% (0.8 °C/s) or 95% (8 °C/s). The addition of P causes a marked drop in the alloy tensile strength due to the precipitation of primary Si, Al_2_Si_2_Sr, and β-AlFeSi hard-phase particles. Therefore, reducing the iron content in the castings may be considered a major objective to be recommended for developing alloys with higher strength and optimum quality values. More than 1000 tensile bars were tested in this study.

## 1. Introduction

The harmful effects of certain metals, such as iron (Fe), which is found in 413 alloys (used in this project), constitute one of the main problems encountered in metallurgy. Indeed, a high percentage of iron promotes the precipitation of Fe phases, which especially reduces ductility [1,2,3,4]. Several authors have suggested the overheating pathway as a means to neutralize the harmful effects of iron without even adding neutralizing elements such as manganese (Mn) and/or chromium (Cr) [5,6]. Certain studies have shown the relationship between the overheating temperature and the iron phases formed, as well as the importance of maintaining low concentrations of such elements as Fe, Si, and Mg while applying a rapid solidification rate, to promote complete precipitation of iron in the form of the less harmful α-AlFeSi Chinese script phase [7,8].

The Al-Fe phase diagram shows a eutectic at the temperature of 655 °C at 1.8% Fe. This eutectic is formed by aluminum (Al) and the phase FeAl_3_, with a maximum solubility of iron in aluminum of 0.052%. Under equilibrium conditions, the phases present in the solid state show a low solubility of silicon in aluminum when a small quantity of iron is added. Under non-equilibrium conditions, four phases can be encountered simultaneously: Al, Si, α-AlFeSi, and β-AlFeSi [9,10].

Intermetallic phases are generally categorized by three morphologies: polygonal crystals, Chinese script, and thin plates. Equilibrium diagrams of diluted Al-Fe-Si alloys include the Θ-AlFeSi (monoclinic) phase, the α-Al_8_Fe_2_Si (hexagonal) phase, the β-Al_5_FeSi (monoclinic) phase, and the α-AlFeSi phase, whose chemical composition is Al_12_Fe_3_Si_2_ (30.7% Fe, 10.2% Si) [8]. These phases dominate during slow cooling, and metastable phases like Al_6_Fe (orthorhombic) and α-Al_20_Fe_5_Si_2_ (cubic) are precipitated during quenching or rapid cooling. In several commercial aluminum alloys that contain Mn or Cr as an impurity, the cubic α-AlFeSi, rather than the hexagonal α-AlFeSi phase, is formed. It was observed that the β-AlFeSi phase precipitates during the liquid–solid (pre-eutectic) reaction, as well as during the formation of the eutectic (co-eutectic) reaction, when the iron concentration exceeds 0.7% [11,12,13].

Increasing the melting temperature (superheating) has been proposed to eliminate the β-AlFeSi intermetallic, which is harmful to the mechanical properties of aluminum alloys. Overheating transforms the β-AlFeSi phase into the Chinese script phase. Overheating and rapid solidification reduce the interdendritic space, the size of the constituents of the eutectic and first phases, and the grain size, despite the increase in real-time solidification [5]. Barlock and Mondolfo [14] add that aluminum alloys contain a germination catalyst (nucleation site), which acts at the zero-supercooling point, and when the overheating exceeds 500 °C, the size and possibly the number of germination particles are reduced but are not eliminated completely.

Crepeau [15] points out that superheating aluminum alloys increases the concentration of hydrogen and oxide inclusions in the molten metal. However, no mention is made of the type of alloy referred to by the author. The mechanism behind contamination of the molten alloy may explain the effect of neutralization and transformation of γ-alumina inclusions into α-alumina. Apparently, the β-AlFeSi phase nucleates on the fine γ-alumina inclusions but not the α-alumina phase [15].

Narayanan et al. [16,17] report that overheating is more effective in refining the α-AlFeSi phase than the addition of manganese in 319 alloys containing 1% iron. They showed overheating is useful not only in transforming the β-phase into Chinese script but also in reducing the size of the intermetallics. Beryllium (Be) is used in Al-Si alloys to reduce oxidation of liquid metal while considering the increase in magnesium (Mg) concentration, and it accelerates hardening processes when the alloy is heat-treated in the T6 condition [18,19]. In addition, beryllium provides alloy fluidity, which increases its castability, especially in the case of automotive alloys. In the presence of beryllium in Al-7% Si-0.3% Mg−0.6% Fe alloys, some authors have observed the formation of a new BeSiFe_2_Al_8_ phase, which has a polygonal shape or Chinese script form [20]. This new phase precipitates mainly within the α-aluminum dendrites, while the β-phase is observed in the interdendritic regions. However, beryllium is used in low concentrations (0.01–0.05%) due to its toxicity [21].

Large primary silicon crystals adversely affect the mechanical properties of Al-Si alloys. Considerable research has been done in an attempt to reduce the size of primary silicon crystals. It has been found that phosphorus (P), when incorporated into the molten metal, can reduce the size of the primary silicon substantially [22]. Phosphorus refining must be conducted in the molten metal during the solidification process since there is no way to refine large primary silicon crystals once they have been formed after casting [23,24,25,26].

Magnesium (Mg) is often used in Al-Si alloys in concentrations ranging from 0.07% to 0.4% to improve the mechanical properties of these alloys through precipitation of the Mg_2_Si phase. When magnesium is added to commercial alloy A319, the β-Al_5_FeSi phase transforms into the π-Al_8_Mg_3_FeSi_6_ phase [26]. The increase in concentration of magnesium increases the volume fraction of this phase. Also, the addition of Mg produces a reduction in the temperature of the silicon eutectic reaction [27,28].

The present investigations were undertaken to focus on the effects of solidification rate and overheating on the microstructure and tensile properties of the hypereutectic alloy A413, taking into consideration the role of additives (Fe, Be, Sr, P, RE), and superheating (melting temperature ~950 °C) on the morphology of β-Al_5_FeSi platelets in the absence of Mn and/or Cr and how both the morphology and characteristics of Fe-intermetallics determine the tensile properties of high Mg (0.35%) 413 aluminum alloys [29,30]. The solidification rates selected correspond to dendrite arm spacings of (DAS) 80–90 µm, 15–20 µm, and 3–5 µm representing, respectively, sand (0.8 °C/s), permanent mold (8 °C/s), and die (50 °C/s) casting techniques [31].

## 2. Experimental Procedure

Different additive elements were incorporated in various combinations and proportions into the base 413.0 alloy, among them Mg, Fe, Be, RE (La), and Sr, which improve the tensile properties of the base alloy 413.0. Additionally, phosphorus (P) was added for refining the primary silicon phase. The alloy was melted in a 28 kg capacity silicon carbide crucible using an electric furnace. The temperature of the metal was brought to ~735 °C and was maintained for the remainder of the melting procedure, including the addition of the various alloying elements, strontium modification, grain refinement, and degassing. Subsequently, the liquid metal was degassed by an injection of pure argon. Chemical analysis was conducted at three different stages for each alloy cast in order to establish a representative concentration sampling of all the elements present in the molten metal. Other batches of liquid metal were superheated at 950 °C for 30 min and then cooled to 735 °C, followed by degassing as above.

The strontium was added in the form of the Al-10% Sr master alloy, whereas the Al-5%Ti-1%B master alloy was used to add titanium diboride. Iron, Be, and P were added in the form of Al-25%Fe, Al-5%Be, and Cu-2%P master alloys, whereas La was added in the form of Al-50%La (using La 99.9% purity). Magnesium and zinc (Zn) were added as pure metals using a perforated graphite bell. Degassing of the liquid metal, when necessary, is done with a rotary graphite stirrer, which rotates at a speed of 150 rpm, and which passes pure dry argon at a flow rate of 0.492 cubic meters per hour for 30 min.

Table 1 lists the chemical composition of the as-received ingots. Two sets of castings were made, one for metallographic investigation of the alloys in the as-cast condition, while the second set was used for tensile testing. As for Be, Mg, and La, due to their high oxidation rate, the initial value was 50% higher than the aimed value to balance the weight loss. It should be emphasized here that Sr, Be, Mg, and La were added when the temperature of the molten metal was about 750 °C, 5 min before the end of degassing i.e., prior to casting. The chemical analysis was conducted using a Spectrolab-JrCCD Spark Analyzer (SPECTRO Analytical Instruments Inc., Mahwah, NJ, USA). The average chemical compositions (three burns per alloy sample) are reported in Table 2. Table 3 lists the chemical compositions of the selected alloys that were used for tensile testing. All alloys were grain-refined with 0.2% Ti in the form of the Al-5%Ti-1%B master alloy.

**Figure 1:**
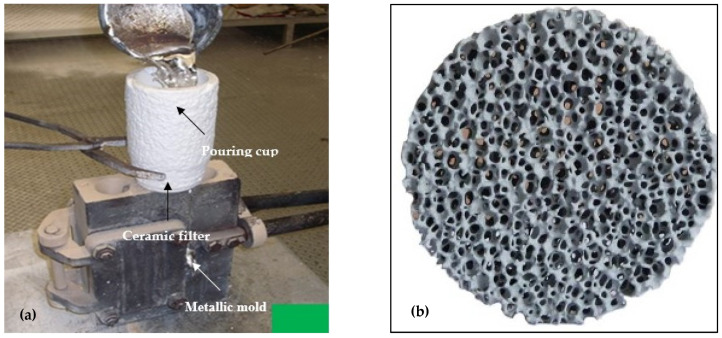
(**a**) Setup used for preparing the tensile test bars. (**b**) A ceramic 20ppi filter is placed at the bottom of the pouring cup, which is made of refractory material. The pouring cup is placed tightly on the top of the sprue of the metallic mold.

**Figure 2:**
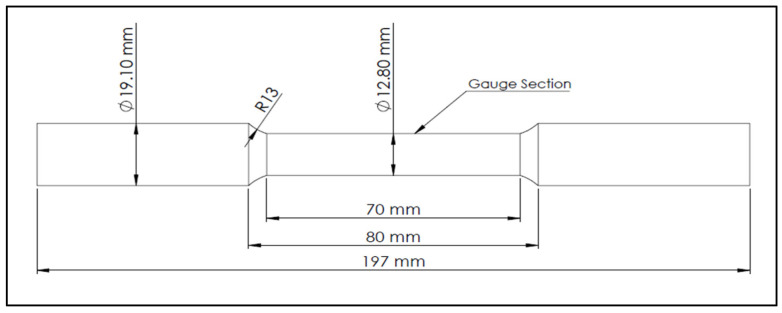
Geometry of the standard tensile test bar obtained from ASTM B-108 permanent mold.

The mold used was a Stahl permanent mold type ASTM B-108 [32]. Figure 1. This mold is symmetrical and allows two tensile test bars to be produced per casting. The interior of the mold is covered with two different coatings: one for the central part of the tensile bars and the other for the rest of the mold. The coating of the central part is graphite-based to allow rapid solidification, while the coating that covers the rest of the mold is made of vermiculite. The mold was preheated at 450 °C prior to casting in an electrical furnace. In order to minimize its formation, the molten metal was poured into the mold through a 20 ppi ceramic filter placed on the top of the sprue using a special set-up, as shown in Figure 1a,b. Figure 2 depicts the geometry of the standard tensile test bar obtained from the ASTM B-108 mold.

In the setting of solutionizing treatment, the aforementioned furnace was pre-programmed according to a cycle such that, for each batch of test specimens, the temperature increased from room temperature to 495 °C in a period of 2 h. The tensile bars were put in at room temperature and ramped to 495 °C. The solution temperature of 495 °C was then maintained for a period of 8 h. Once the solution treatment was completed, the specimens were quenched from the temperature of 495 °C to room temperature. The setup for conducting the water quenching consists of a tank of water, which has a temperature that is close to 60 °C, and into which the test pieces taken out of the furnace were immersed.

Once cooling by quenching was complete, the test pieces were reheated in the furnace according to one of the five chosen artificial aging treatments (temperatures and times pre-programmed using the same programmable furnace). These methods of artificial aging consisted of taking turns to bring the temperature of a batch of test pieces from room temperature to 155 °C in half an hour. The artificial aging temperature was maintained for a period of 5 h. Cooling, at the end of artificial aging, was then conducted in ambient air. Subsequently, tensile tests were conducted on the test bars of selected alloys, where the as-cast bars were solution heat-treated and then artificially aged as per the five selected aging treatments.

The tensile specimens were tested using an MTS Servohydraulic Mechanical Testing machine (MTS Systems, Eden Prairie, MN, USA) at a strain rate of 4 × 10^−4^/s. The data were collected by a data acquisition system connected to the machine. Conversion software processes this data to transform elongation into strain and force into stress. The software numerically determines the elastic limit (YS), the ultimate tensile strength (UTS), and the %Elongation to failure. The latter was obtained using a 2-inch extensometer attached to the gauge length of the bar (also supplied by MTS Systems).

Thermal analyses were conducted on the ingots of the alloys studied. Each experiment involved melting the alloy sample at ~725 °C and pouring the liquid metal into a graphite mold preheated at 600 °C. A type-K (chromel-alumel) thermocouple was installed through a hole at the bottom of the graphite mold, reaching the center of the mold and connecting to a computer using the installed software that recorded the temperature-time data. The solidification curve and its first derivative could then be plotted upon pouring the molten metal into the preheated mold. The solidification curves describe the evolution of the solidification temperature of the alloys studied as a function of time, and the first derivative curves describe the stages of formation of the various phases present in these alloys.

The observations of the microstructure were conducted with an Olympus BH2UMA optical microscope (Supplier: Evident Canada Inc., Québec, Quebec G1P, Canada) as well as with a JEOL JXA-8900L WD/ED (JEOL USA Inc., Peabody, MA, USA) electron microprobe that was operated at a voltage of 20 KV and a current of 30 Nano amperes. The latter made it possible with the backscattered electron imaging mode to obtain high magnification images of the microstructure while using energy dispersive X-rays (EDX) and wavelength dispersion spectroscopy (WDS) to analyze the chemical compositions of different phases.

## 3. Results and Discussion

### 3.1. Characteristics of β-Al_5_FeSi Platelets

Table 4 documents the combined effects of both the solidification rate and alloy composition on the characteristics of β-Al_5_FeSi platelets, including average platelet length, average platelet thickness, platelet density, and average total surface area/cm^2^. Increasing the solidification rate in terms of a DAS of about 80–90 µm obtained with the preheated graphite mold casting to 15–20 µm when using the permanent metallic mold reduced the average platelet length by about 82%. Further reduction to about 98% was achieved when the molten metal was poured directly into water (water quench in Table 4) in the form of droplets (the solidified droplets provided a DAS of about 3.5 µm).

Modification with 195 ppm Sr proved to be effective in further reducing the β-platelet average length by about 70% for the hot graphite mold, whereas a combined high solidification rate (metallic mold) and Sr treatment resulted in bringing down the platelet length by about 96%.

Apparently, the addition of Be together with Sr is capable of reducing the β-platelet length by 87% and by 96% for the hot graphite mold and metallic mold, respectively. The addition of Ti or Zn in place of Be resulted in the same effect. It is evident from the platelet density that all melt treatments caused marked fragmentation of the initial length of the β-platelets. Another point that may be considered is that P, or more precisely, AlP particles, may act as nuclei for the precipitation of the platelets. However, this process is associated with a high fluctuation in the measurements (standard deviation). It should be kept in mind that all these alloys were superheated at 950 °C prior to casting from 750 °C.

**Figure 3:**
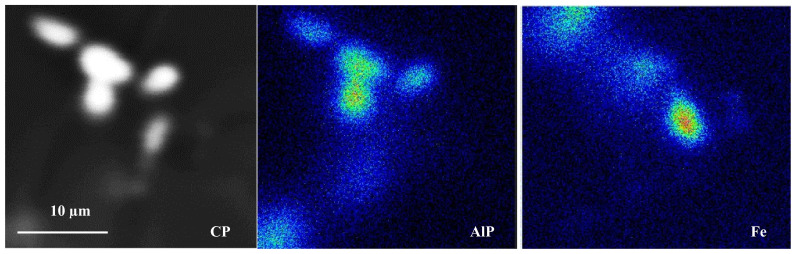
Precipitation of Fe compounds on the AlP particles in EFPHT alloy.

Grain refining using the Al-5%Ti-1%B master alloy was recommended by several authors as an effective method for improving the alloy strength [33,34,35,36,37]. Table 5 highlights the role of grain refiner on the fragmentation of β-platelets in the present study. As may be seen, the introduction of TiB_2_ to the molten metal followed by superheating treatment resulted in a significant decrease in the length of the platelets, and the reaction was more effective when the alloy was grain refined and Sr-modified. In addition, the difference in platelet length between superheated alloys and those poured directly from 750 °C is about ± 20% [38,39]. Figure 3 depicts the precipitation of an Fe-rich phase on the AlP particles in the EFPHT alloy sample.

### 3.2. Microstuctural Characterization

**Figure 4:**
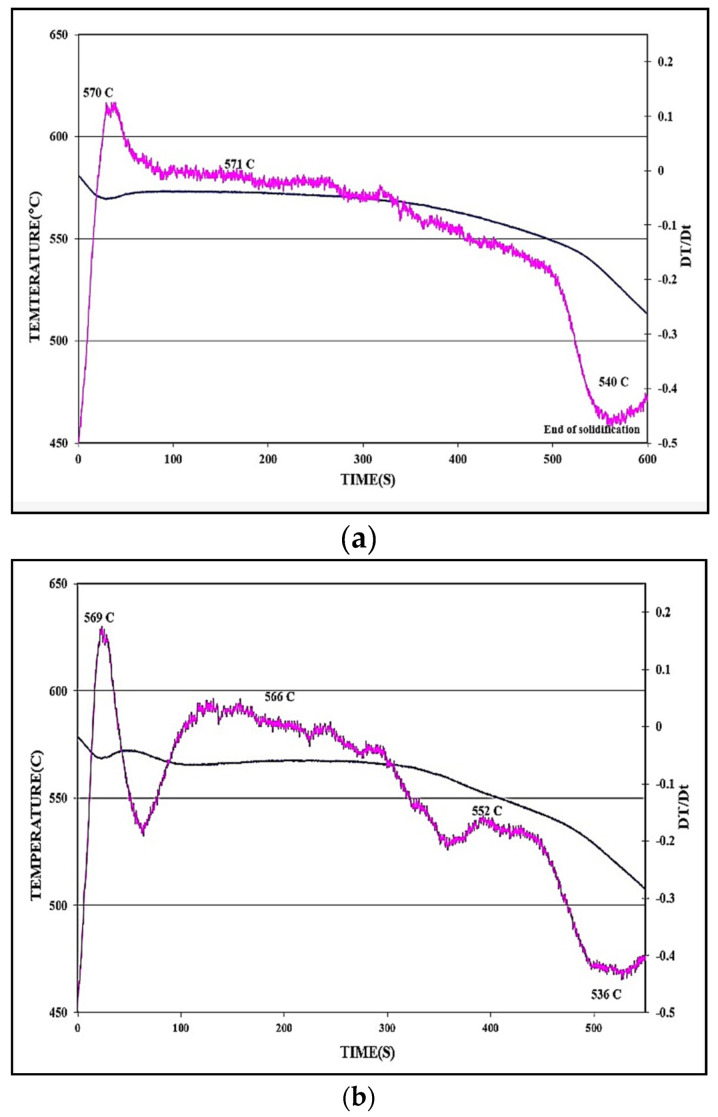
Temperature-time solidification curves and their first derivatives (in fuschia color) for: (**a**) non-modified, and (**b**) Sr-modified 413 alloy. Alloys were cast in the 600 °C preheated graphite mold.

**Figure 5:**
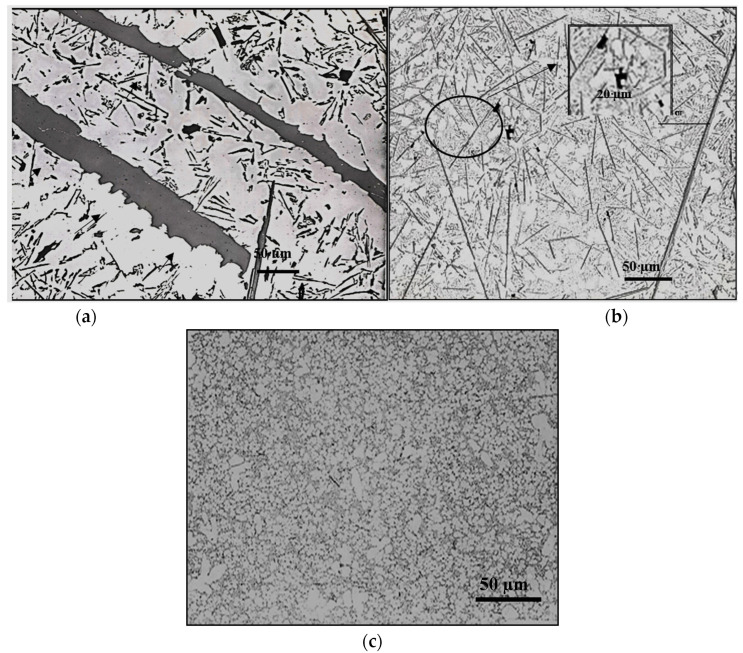
Effect of solidification rate on the size and distribution of β-platelets in: (**a**) hot graphite mold sample (DAS ~90 µm); black arrows show necking prior to fragmentation; (**b**) metallic mold sample (DAS ~20 µm); (**c**) solidified droplet from direct quench in cold water (DAS ~3 µm). Inset in (**b**) shows precipitation of microporosity on the surfaces of β-platelets.

Figure 4 illustrates the solidification curves of 413.0 alloy in the non-modified (Figure 4a) and Sr-modified (Figure 4b) conditions, respectively. Based on Figure 4, the melting point of the base alloy is about 570 °C. Modification with Sr has no effect on either the melting temperature or the solidification time of ~550–600 s. Table 6 summarizes the details of the solidification curves presented in Figure 4 in terms of the different parameters involved. In the present section, the EF alloy contains about 1.2% Fe, which is close to the Fe content in high-pressure die-casting alloys such as the B380.1 alloy [40,41,42]. As seen in Table 4, with the increase in solidification rate, the average length of the β-platelets decreased from about 400 µm to ~7 µm, with the decrease in the DAS from 90 µm to ~3 µm, as shown in Figure 5.

**Figure 6:**
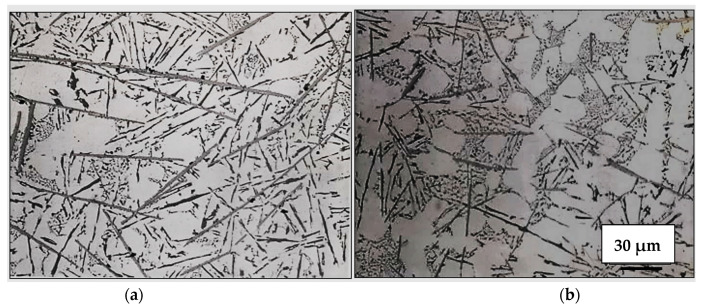
Effect of direct pouring temperature on the size and distribution of β-platelets: (**a**) 750 °C, (**b**) 1050 °C. Samples were cast in the metallic mold.

Pouring liquid metal from a temperature as high as 1050 °C produced a significant reduction in the length of the β-platelets to ~40 µm from about 250 µm for samples poured directly from 750 °C, due to enhanced rates of β-phase decomposition from Al_5_FeSi to Al_6_Fe [42], coupled with dissolution in the liquid metal, as seen in Figure 6. However, the application of such high temperatures will not allow for degassing using the graphite impeller, as described in the experimental procedure section.

**Figure 7:**
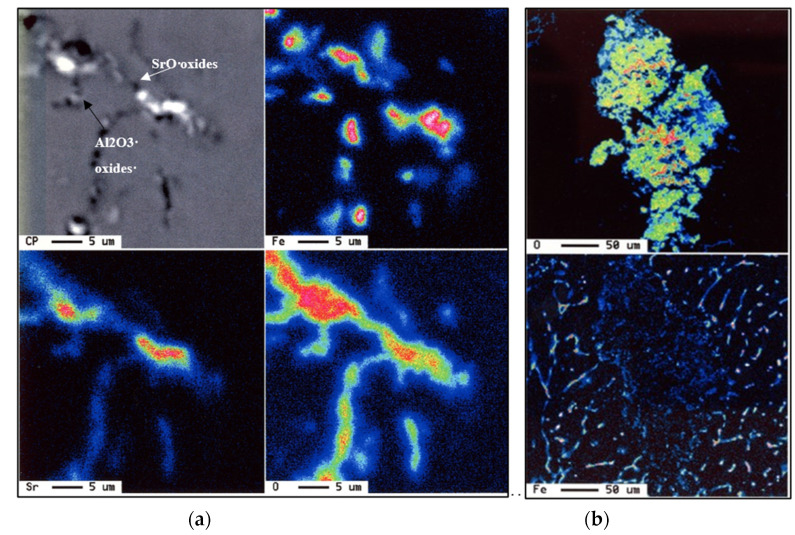
(**a**) Oxide films associated with samples poured directly from 1050 °C, showing precipitation of Fe intermetallic on the edges of the oxide film, (**b**) precipitation of Fe-containing phase particles on the edges of an oxide film.

**Figure 8:**
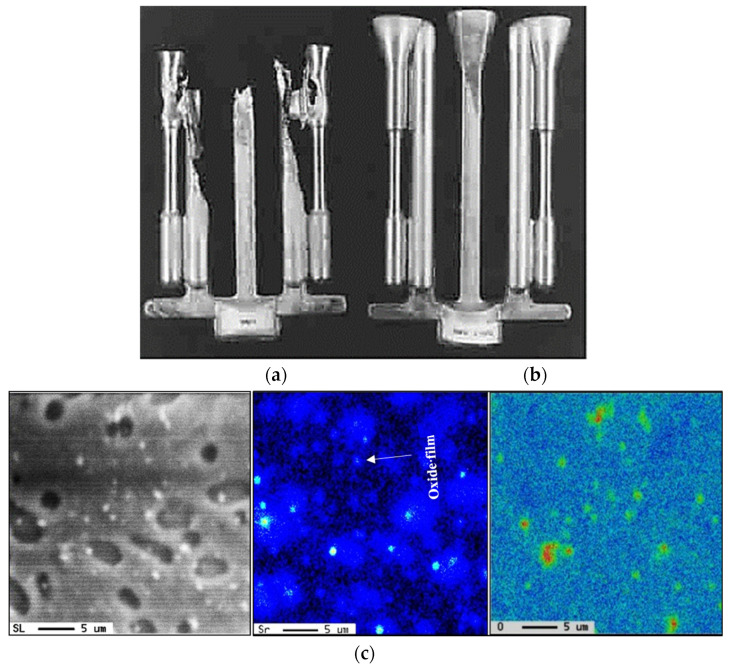
(**a**) Pouring temperature about 1050 °C–incomplete casing, (**b**) pouring temperature 750 °C–complete casting, (**c**) size and distribution of SrO in samples poured from 750 °C following degassing for 30 min using pure dry argon gas.

According to the Ellingham diagram [43], at such high temperatures, Al, Mg, and Sr have a high affinity to react with oxygen, forming different oxides i.e., Al_2_O_3_, MgO, and SrO, as depicted in the microstructure shown in Figure 7, which reveals massive aluminum oxide films (black arrow) and SrO (white arrow). These oxides are suitable sites for precipitation of β-platelets and would significantly deteriorate the alloy strength [44,45,46]. In contrast, for samples cast from 750 °C, Figure 8 exhibits scattered SrO spots rather than patches as seen in Figure 7. Although Sr was added to the molten metal just before pouring, it is impossible to stop the formation of SrO. Another problem encountered with pouring directly from 1050 °C is excessive fluidity of the liquid metal, leading to leaking of the metal out of the mold, resulting in an incomplete casting, as shown in Figure 8a, compared to the casting with molten metal poured from 750 °C, as depicted in Figure 8b. Figure 8c depicts the precipitation of SrO oxide in the form of fine dispersed particles/patches when the molten metal was poured from 750 °C and passed through a ceramic filter prior to entering the mold, as was shown in Figure 1.

**Figure 9:**
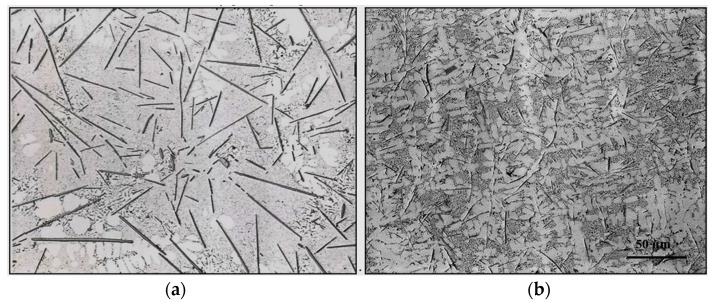
Combined effects of solidification rate, Sr modification, and addition of Be on the size and distribution of β- platelets: (**a**) graphite mold, (**b**) metallic mold. Both melts were superheated at 950 °C.

The combined effect of adding both Sr and Be, coupled with the solidification rate on the size and distribution of the β-platelets, is presented in the optical micrographs of Figure 9. As can be observed, the use of Be has enhanced the fragmentation and dissolution process of the β-platelets, leading to a significant reduction in their length from about 150 µm (Figure 9a) to about 10–15 µm (Figure 9b). As mentioned previously in the introduction section, Fe reacts with Be, forming a complex compound, i.e., the BeSiFe_2_Al_8_ phase, which would lower, to some extent, the concentration of free Fe. Hence, the amount of active Fe to form the β phase would be less than the added Fe.

**Figure 10:**
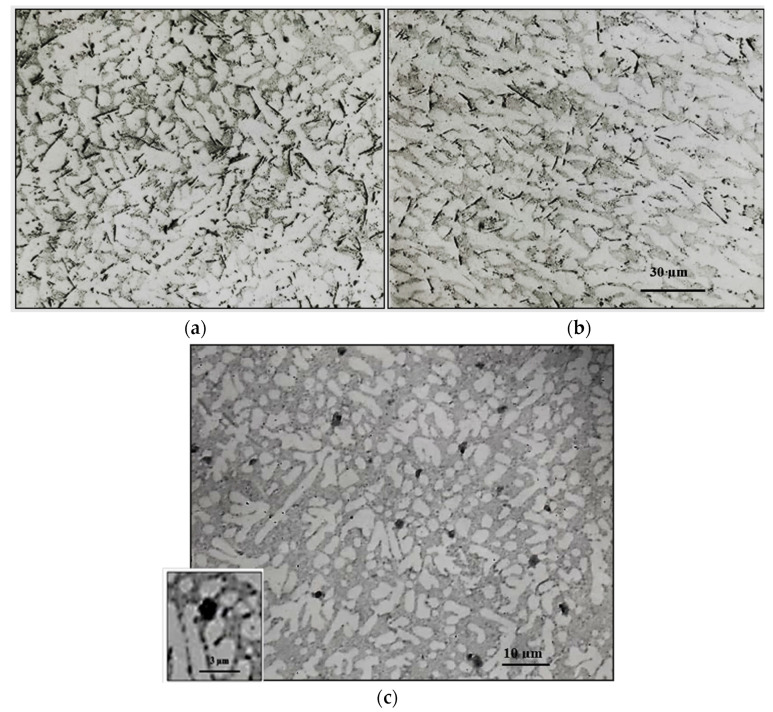
Effect of added TiB_2_ or Zn to the Sr +Be-containing alloy on the size and distribution of β-platelets: (**a**) TiB_2_ (0.2%Ti)—metallic mold sample, (**b**) 1%Zn—metallic mold sample, (**c**) Sr+Be-containing alloy—solidified droplet (water quenching method). All samples were superheated at 950 °C and poured from 750 °C.

**Figure 11:**
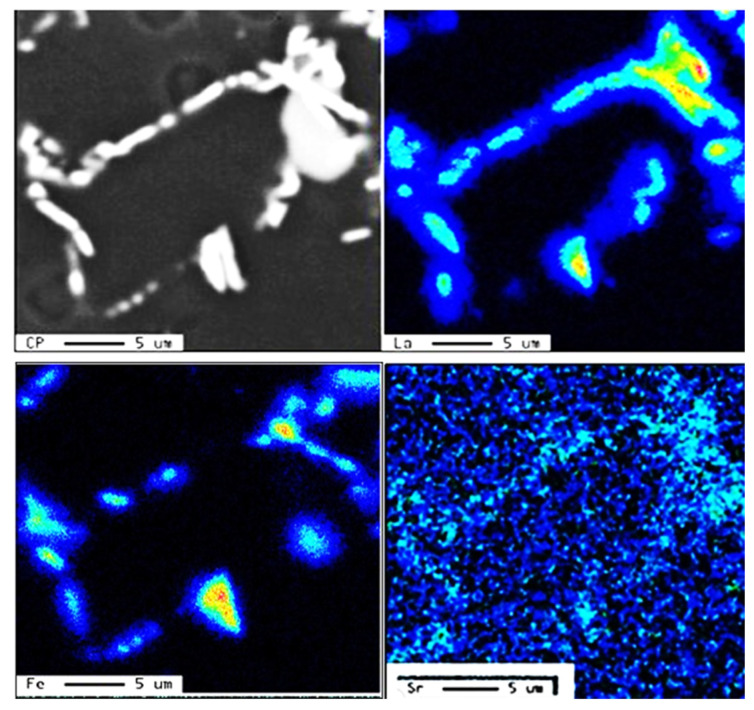
Precipitation of β-phase particles in La-containing Sr-modified 413 alloy (metallic mold).

Figure 10 illustrates the role of added Ti (in the form of TiB_2_, Figure 10a), or 1% Zn (Figure 10). In Figure 10b, the same alloys shown in Figure 9 are observed accelerating the rates of fragmentation of the β-platelets. It is evident that none of these additions brought about marked changes above those displayed in Figure 10b. In other words, the main role of using Ti or Zn is grain refining or accelerating the hardening rate, respectively. In other words, the main role of using Ti or Zn is grain refining or accelerating the hardening rate, respectively. Another aspect to be highlighted is the spheroidization of the β-platelets when applying the water quenching method, as depicted in Figure 10c. Figure 11 illustrates the role of La as an active site for β-phase precipitation.

### 3.3. Tensile Properties

**Figure 12:**
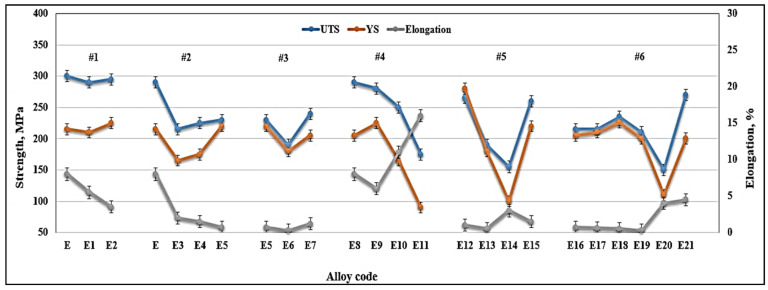
Variation in the alloy tensile parameters as a function of alloy composition.

Figure 12 depicts the effects of the various alloying elements on the tensile properties of the selected alloys (see Table 3) in the T6 condition. The contribution of these additives to the base alloy E is summarized in Table 7 (details for the calculation of ΔP are presented in Appendix A). As highlighted in Figure 12, the addition of Be or P to the E (413.0 + 0.35%Mg) alloy resulted in a slight increase in both UTS and YS, with decreases in the alloy ductility (Series #1) due to partial conversion of the β-platelets into the compacted BeSiFe_2_Al_8_ phase, or fragmentation of the platelets, resulting in the formation of the Al_6_Fe phase.

Series #2 represents the influence of increasing the concentration of Fe to 1.8% total. Increasing the Fe content from 0.4% in E0 alloy to 0.8% caused a drop in the UTS, YS, and %El levels by about 30 MPa, 83 MPa, and 5.5%, respectively, followed by a marginal increase in these parameters, with a further increase in Fe added to ~1.8% in total. This may be attributed to the hardening effect of the fragments of β-platelets, as shown in Figure 5b, similar to the behavior of metal matrix composites [47,48,49,50,51,52].

**Figure 13:**
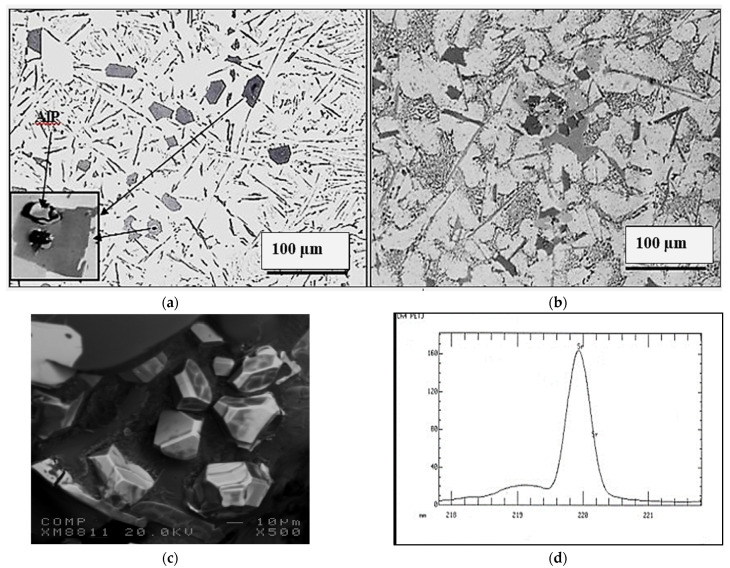
Effect of P addition on the precipitation of: (**a**) primary Si particles (non-modified alloys), (**b**) precipitation of Al_2_Si_2_Sr phase particles in Sr-modified alloys, (**c**) an example of 3D shape of Sr-containing precipitates, (**d**) scan through (**c**).

The addition of P to the alloy melt results in the precipitation of a large number of AlP particles (see Figure 13). The resulting increase in density of the β-platelets (see Table 7) will eventually ruin the alloy mechanical properties, particularly in alloys with a high Fe content, such as the E6 alloy, causing a significant drop in the values of UTS, YS, and %El by 205 MPA, 110MPa, and about 6%, respectively. Although the addition of Be (E7 alloy) revealed a tendency to improve the alloy tensile parameters, the values of UTS and YS were 50–100 MPa below the levels obtained for the E alloy.

Series #4 and #5 highlight the beneficial effect of modifying the alloys with Sr in accelerating the fragmentation rates of the β-platelets, as shown in Figure 10b, which, in turn, would contribute to the alloy strength. The effect of strontium as a modifier is attenuated by the presence of phosphorus, which reacts with it. Thus, alloys containing high concentrations of phosphorus require greater amounts of strontium to produce an acceptable microstructure.

**Figure 14:**
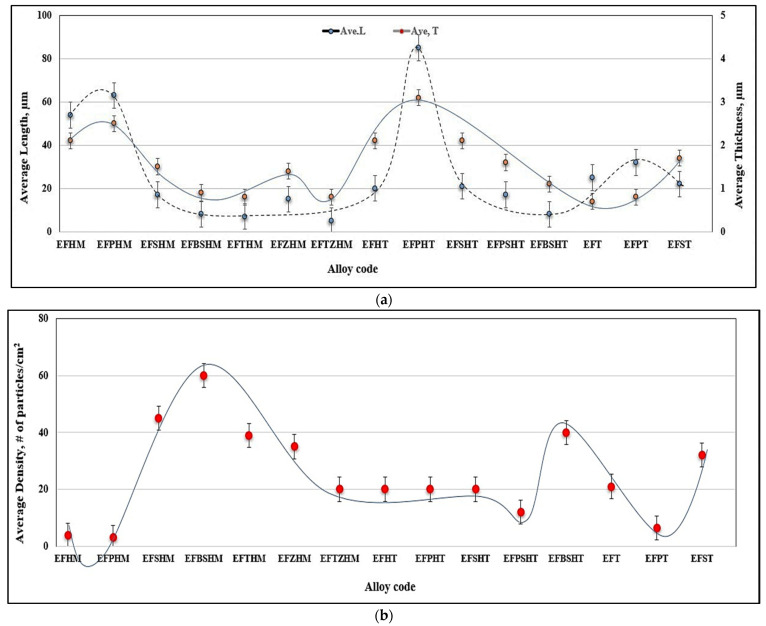
(**a**) Effect of additives and superheating on the average length and average thickness of β- platelets—samples obtained from metallic mold castings; see Table 4 and Table 5 for details. (**b**) Effect of additives and superheat on the density of β-platelets.

Large primary silicon crystals adversely affect the mechanical properties of Al-Si alloys [53,54] e.g., as in the case of the E11 alloy. Figure 13a illustrates the precipitation of primary Si particles on AlP particles (inset micrograph). In this case, the precipitation of primary Si is followed by the eutectic reaction. However, in the E9 alloy, containing 320 ppm Sr, crystals of Al_2_Si_2_Sr phase particles (grey color) precipitate all over the matrix, as seen in Figure 13b. Considering the high hardness of Si (7 Mohs), these particles would contribute to the alloy strength [55], counteracting the negative effect of 1.8%Fe in the E15 alloy along with the presence of Be. Nevertheless, the degradation effect of P could not be overcome by high Sr addition, bringing the alloy tensile parameters far below those offered by the base E alloy (i.e., ΔP values of −150 MPa (UTS), −120 MPa (YS), and −22%El, taking the base E alloy (ΔP = 0) as the reference line). According to Gao et al. [56] and Barrirero et al. [57], the treatment of Al-Si alloys with Sr and the formation of the Al_2_Si_2_Sr phase would neutralize the nucleation of Si, reducing the frequency of their precipitation. The data presented in Table 4 and Table 5 showing the effect of superheating and additives on the dimensions of β-platelets are plotted in Figure 14a. As can be seen, the addition of P results in the precipitation of massive β-platelets, causing major deterioration of the tensile properties. Although Be is toxic, careful use will counteract the negative effect of P. Figure 14b depicts the variation in the density of β-platelets as a function of types of additives and superheat. As can be observed, the behavior of particle density is opposite to that noted for the average length and thickness parameters of the β-platelets displayed in Figure 14a.

**Figure 15:**
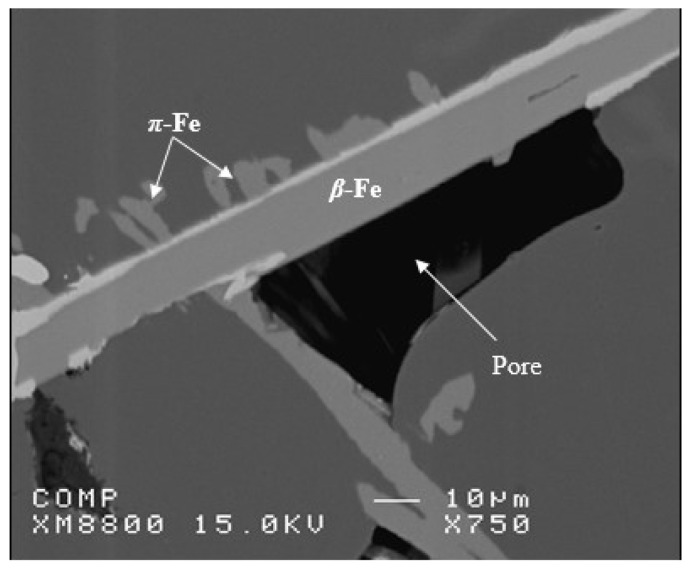
An example of reduction in the molten metal circulation by long intercepting β-platelets in 1.8%Fe-containing alloys solidified at 0.8 °C/s.

Another point to consider is the role of superheating. Series #6 shows that alloys E16 to E18 are characterized by their brittleness, with their ductility reaching about 0.5% (compared to 15% for alloy E), whereas alloy E19 revealed some recovery in ductility (about 5%). As in the other cases discussed above, the addition of 500 ppm Be proved to be very effective in counteracting the loss in the alloy tensile strength properties (ΔP values of −25 MPa (UTS), 10MPa (YS), and −17%%El). One of the main reasons for the observed decrease in alloy strength in high Fe-content (~1.8%) alloys is the porosity formation caused by the restriction of fluid motion to fill up the gap between the intercepting long β-platelets, as illustrated in Figure 15.

### 3.4. Alloy Quality: Interpretation of Tensile Properties Using the Quality Index

The quality of aluminum alloy castings may be defined using numerical values, which correlate with their mechanical properties. Drouzy et al. [58,59] first proposed these numerical values in 1980 and termed them quality indices. These may be represented by the following equation:

(1) $$Q\;=\;\;\sigma_{uts}\;+\;d\;\log\;(E_{f})$$

where *Q* is the quality index in *MPa*; $\sigma_{uts}\;$ refers to the ultimate tensile strength in *MPa*; *E_f_* refers to the percentage elongation to fracture; and *d* is a material constant equal to 150 MPa for Al-7Si-Mg alloys. The probable yield strength, σ*_P_*_(*YS*)_, for the same alloy may be proposed as:

(2) $$\sigma_{P(YS)}=a\;\sigma_{UTS}\;-\;b\;\log\;(E_{f})\;+\;c$$

where the coefficients *a*, *b*, and *c* for Al-7Si-Mg alloys were determined as 1, 60, and −13, respectively, with the constants *b* and *c* expressed in units of MPa.

**Figure 16:**
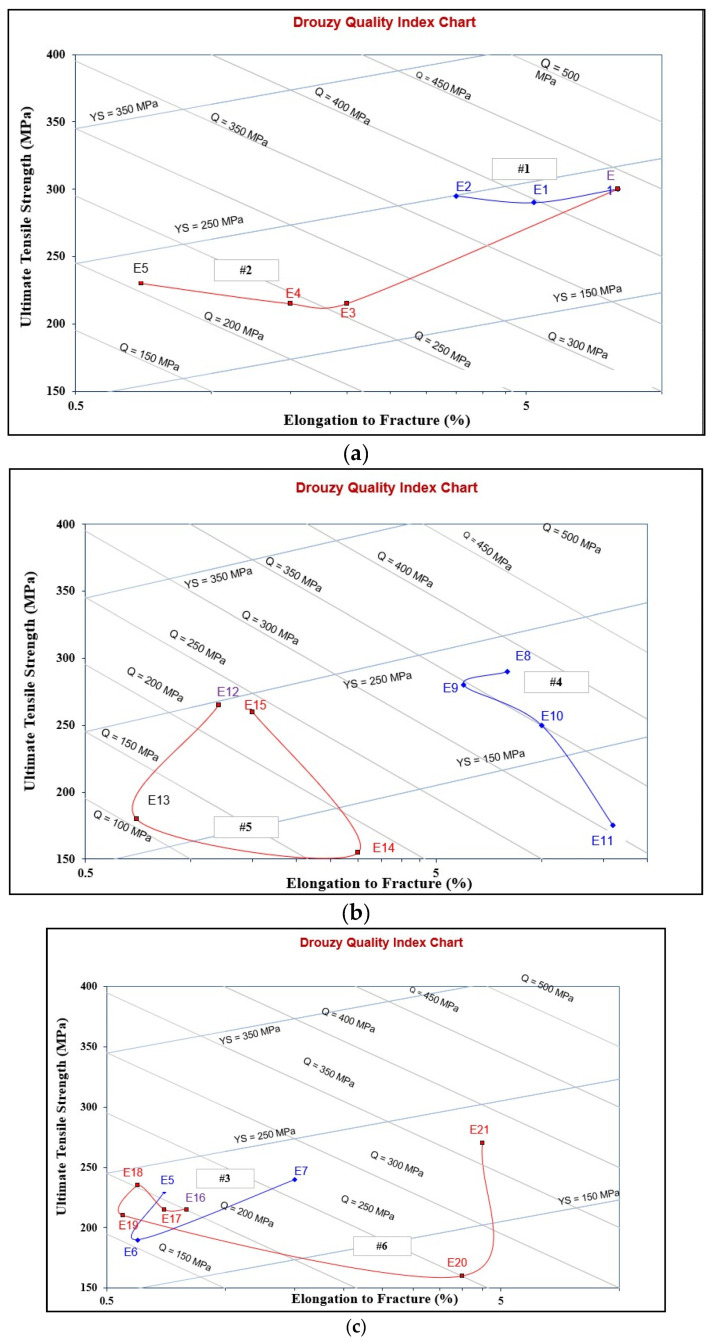
Quality values of the alloys used for tensile testing, tested in the T6-tempered condition: (**a**) Series #1 and #2, (**b**) Series #4 and #5, (**c**) Series #3 and #6.

Figure 16a presents the Quality Index chart as proposed by Drouzy et al. [58,59], showing the Q values for the Series #1 and Series #2 alloys. A significant drop in quality of the E alloy is noted once the Fe content is increased to 0.8% (from 430 MPA to about 250 MPa), whereas increasing the Fe concentration to 1.8% resulted in a marked decrease in the alloy ductility and, hence, its Q-value or quality (~200 MPa).

**Figure 17:**
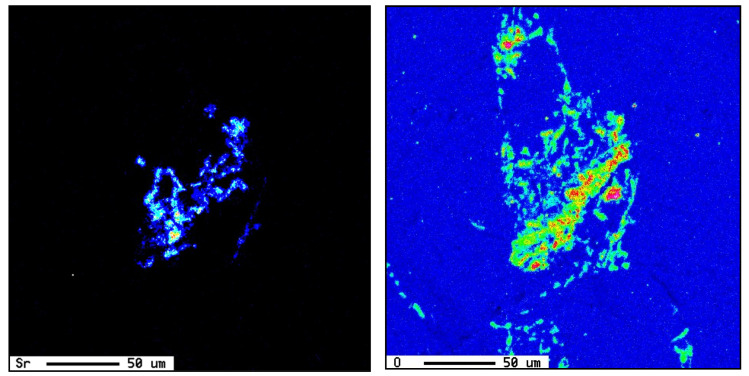
Formation of SrO in E8 alloy.

Figure 16b illustrates the beneficial effect of modification with the right amount of Sr, as in the case of alloy E8, which achieves a Q-value of about 380 MPa compared to 430 MPa for the non-modified E alloy. The difference may be brought about by the porosity caused by the formation of SrO, as exemplified in Figure 17. Over-modification with 320 ppm Sr, coupled with the precipitation of Al_2_Si_2_Sr phase particles (shown in Figure 13b), resulted in a decrease of the Q-value of the E8 alloy by about 30 MPa for the E9 alloy.

The use of 30 ppm P and its ability in increasing the volume fraction of primary Si and nucleation of Al_2_Si_2_Sr particles led to a reduction in the UTS level. However, there was no marked effect on the Q-values due to the improvement in the alloy ductility.

When increasing the Fe content to 1.8%, Sr addition increased the Q-index to about 230 MPa, which is very close to that achieved by the E5 alloy and E15 alloy containing 500 ppm Be. The worst values were noted for the P-containing alloys, as seen in Figure 16b. Figure 16c reveals the effect of superheating on the Q-values of 1.8%Fe-containing alloys, showing a significant enhancement in the value of Q, reaching 370 MPa in the case of E21 alloy, compared to the untreated E5 alloy.

## 4. Conclusions

The current investigations were performed with a view to highlight the role of a number of metallurgical parameters on the tensile properties and quality indices of high-strength eutectic Al-11.7%Si-0.35%Mg-based alloys. The studied variables are: iron level; solidification rate; Sr Be, P, Ti, and Zn addition. Quality charts were used for selecting the optimum conditions to be applied in the industry to achieve high strength and optimum quality in Al-Si castings. From the results obtained, the following salient points may be listed:

1. Iron has a marked degradation impact on both the strength and the quality of the 413 based alloy castings due to the size and morphology of the β platelets. Tolerable Fe concentrations and methods of neutralization/fragmentations or dissolution have been discussed.
2. The addition of Sr plays a significant role in improving the strength of these alloys. The combined addition of Sr and Be enhances the alloy performance due to destabilization of the β-Al_5_FeSi platelets, and their consequent fragmentation, in addition to a partial transformation of the Al_5_FeSi platelets to the new BeSiFe_2_Al_8_ phase, which has a polygonal shape or Chinese script form.
3. Applying superheating at 950 °C would reduce the average β-platelet length by about 95%, which facilitates their dissolution thereafter during the solutionizing treatment. Also, over-modification with Sr results in the precipitation of Al_2_Si_2_Sr polygonal particles, causing reduction in the alloy strength.
4. The addition of P has a strong tendency to deteriorate the alloy tensile properties and quality indices caused by accelerating the precipitation of Fe-containing intermetallic and primary Si and Al_2_Si_2_Sr phase particles, reducing the alloy ductility to about 0.6% from an initial value of 15% for the base alloy.
5. The addition of Ti or Zn has no effect on Fe-phase precipitation or dimensions.
6. Increasing the solidification rate to about 50 °C/s would lead to complete dissolution of 1.8%Fe in the aluminum matrix.

## Figures and Tables

**Table 1:** Chemical composition of as received ingots of 413.0 alloy (coded E0).

Alloy	Elements (wt.%)									
	Si	Fe	Cu	Mn	Mg	Cr	Ti	Be	Sr	Al
E0–1	10.47	0.3801	0.4734	0.2374	0.0410	0.0302	0.0431	0.0006	0.000	87.8
E0–2	10.69	0.3561	0.4968	0.2462	0.0487	0.0291	0.0645	0.0002	<0.0000	87.8
E0–3	10.87	0.3593	0.4972	0.2424	0.0477	0.0295	0.0648	0.0002	0.0001	87.7

**Table 2:** Compositions of alloys prepared for metallographic examination.

Code	Description
EFHG EFPHG EFSHG EFBSHG	EF * alloy, super heating, graphite mold EF alloy + 30 ppm P, super heat, graphite mold EF alloy + 195 ppm Sr, super heat, graphite mold EF alloy + 195 ppm Sr + 500 ppm Be, super heat, graphite mold
EFHM EFPHM EFSHM EFBSHM EFTHM EFZHM EFTZHM EFSLHM	EF * alloy, super heating, metallic mold EF alloy + 30 ppm P, super heat, metallic mold EF alloy + 195 ppm Sr, super heat, metallic mold EF alloy + 195 ppm Sr + 500 ppm Be, super heat, metallic mold EF alloy + 0.2%Ti, super heat, metallic mold EF alloy + 1%Zn, super heat, metallic mold EF alloy + 1%Zn + 0.2%Ti, super heat, metallic mold EF alloy + 195 ppm Sr + 0.5%La, super heat, metallic mold
EFHC EFPHC EFSHC EFBSHC	EF * alloy, super heating, water quenching EF alloy + 30 ppm P, super heat, water quenching EF alloy + 195 ppm Sr, super heat, water quenching EF alloy + 195 ppm Sr + 500 ppm Be, super heat, water quenching

* EF alloy = E0 with 1.2%Fe total.

**Table 3:** Chemical compositions of selected alloys for tensile testing.

Alloy Codes	Composition
E	E0 + 0.35% Mg
E1	E + 0.5%Be
E2	E + 0.0030%P
E3	E, 0.8%Fe total
E4	E, 1.2%Fe total
E5	E, 1.52%Fe total
E6	E5 + 30 ppm P
E7	E5 + 0.05%Be
E8	E + 195 ppm Sr
E9	E + 380 ppm Sr
E10	E + 195 ppm Sr + 30ppm P
E11	E + 380 ppm Sr + 30ppm P
E12	E5 + 195ppm Sr
E13	E5 + 195 ppm Sr + 30ppm P
E14	E5 + 380 ppm Sr + 30ppm P
E15	E5 + 380 ppm Sr + 0.05%Be
E16	E5-superheated
E17	E12-superheated
E18	E14-superheated
E19	E6-superheated
E20	E13-superheated
E21	E15-superheated

**Table 4:** Effect of mold type on post-dendritic β-Al_5_FeSi platelet characteristics.

Mold Type/DAS *	Alloy Code	Av. Length (µm)	Av. Thickness (µm)	Density (#/cm^2^)	Av. Surface Area (µm^2^/cm^2^)
Graphite mold DAS 80–90 µm	EFHG EFPHG EFSHG EFBSHG	389 ± 6 174 ± 3 124 ± 4 52 ± 3	12.3 10.4 3.4 3.0	1.1 × 10^3^ 5.2 × 10^3^ 6.2 × 10^3^ 2.9 × 10^4^	1.1 × 10^7^ 6.0 × 10^7^ 8.7 × 10^6^ 9.4 × 10^6^
Metallic mold DAS 15–20 µm	EFHM EFPHM EFSHM EFBSHM EFTHM EFZHM EFTZHM	54 ± 2 63 ± 3 17 ± 7 8 ± 5 7 ± 3 13 ± 4 5 ± 2	2.1 2.4 1.5 0.9 0.8 1.4 0.8	3.9 × 10^4^ 3.1 × 10^4^ 4.5 × 10^5^ 5.9 × 10^4^ 3.9 × 10^5^ 3.6 × 10^5^ 2.7 × 10^5^	3.6 × 10^6^ 5.7 × 10^6^ 2.5 × 10^6^ 7.5 × 10^5^ 3.5 × 10^5^ 1.1 × 10^5^ 2.8 × 10^5^
Water quench (solid droplet) DAS = 3–5 µm	EFHC EFPHC EFSHC EFBSHC	7 ±3 9 ± 5 Nil Nil	0.2 0.2 Nil Nil	4.5 × 10^3^ 1.2 × 10^4^ Nil Nil	6.3 × 10^3^ 2.2 × 10^4^ Nil Nil

* DAS: dendrite arm spacing.

**Table 5:** Effect of solidification rate and grain refiner on the characteristics of β-platelet-metallic mold.

Superheating Temperature, (°C)	Alloy Code	Av. Length (µm)	Av. Thickness (µm)	Density (#/cm^2^)	Av. Total Surface Area (µm/cm^2^)
950 °C	EFHM	54 ± 18	2.1	3.9 × 10^4^	3.6 × 10^6^
950 °C	EFHT EFPHT EFSHT EFPSHT EFBSHT	20 ± 1 91 ± 7 20 ± 2 17 ± 9 8 ± 2	1.5 2.1 1.6 1.1 0.7	2.0 × 10^5^ 2.1 × 10^4^ 2.1 × 10^5^ 2.0 × 10^5^ 4.6 × 10^5^	6.0 × 10^6^ 4.0 × 106 7.0 × 10^6^ 3.7 × 10^6^ 3.3 × 10^6^
750 °C	EFT EFPT EFST EFPST EFBST	25 ± 5 32 ± 2 22 ± 1 22 ± 1 12 ± 8	0.8 1.7 0.8 1.1 0.7	2.0 × 10^5^ 6.3 × 10^4^ 2.1 × 10^5^ 1.2 × 10^5^ 2.4 × 10^5^	4.0 × 10^6^ 3.4 × 10^6^ 3.7 × 10^6^ 4.0 × 10^6^ 1.1 × 10^6^

**Table 6:** The effect of addition of Sr on the eutectic undercooling of 413 alloy, as seen in Figure 4.

Alloy	Undercooling Parameters Related to the Al-Si Eutectic Reaction					
	Te1 (°C)	Te2 (°C)	ΔTe (°C)	te1 (s)	te2 (s)	Δte (s)
413 (Figure 4a)	570.1	571.3	1.4	124.4	233.6	132.2
431 + Sr (Figure 4b)	565.6	568.3	2.7	104.4	213.2	88.8

**Table 7:** Values of ΔP (P = Property) for the six series of alloys selected for tensile testing.

Alloy	ΔP−UTS (MPa)	ΔP−YS (MPa)	ΔP−%EL
**Series #1**			
E	0	0	0
E1	−10	−5	−2.5
E2	−5	10	−4.5
**Series #2**			
E	−10	0	0
E3	−85	−40	−6
E4	−80	−35	−6.5
E5	−70	5	−7.3
**Series #3**			
E5	−70	5	−7.3
E6	−110	−35	−7.7
E7	−60	−10	−6.8
**Series #4**			
E8	−10	−10	0
E9	−20	10	−2
E10	−50	−50	3
E11	−125	−125	8
**Series #5**			
E12	−35	65	−7
E13	−110	−35	−7.5
E14	−145	−115	−5
E15	−40	5	−6.5
**Series #6**			
E16	−85	−10	−7.3
E17	−85	−5	−7.4
E18	−65	11	−7.5
E19	−90	−15	−7.7
E20	−150	−105	−4
E21	−30	−15	−3.5

## Data Availability

The original contributions presented in the study are included in the article, further inquiries can be directed to the corresponding authors.

## Appendix A

A comparison of the tensile properties of the selected alloys studied in this investigation, using various additives and amounts, was conducted using a system where a comparison was made in terms of the difference in properties obtained using the base alloy as the reference alloy.

This system consists of observing the difference between the properties of an alloy and those of the base alloy 413.0 (coded E alloy), denoted by ΔP, where P represents a property (P = UTS, YS, and %EL in the present case). The ΔP values presented in Table 6 were obtained by taking the base E alloy (ΔP = 0) as the reference line.

The ΔP values were calculated as follows:

UTSx − UTSi = ΔUTSx

In this formula, UTSx represents the ultimate limit (UTS) of the alloy with one or more new elements added to the base alloy. UTSi represents the ultimate limit of alloy I; and ΔUTSx is the difference between the two, which will serve as a comparison tool. The same calculation was also applied for the elastic limit (YS) and for the percentage elongation (%EL). Accordingly, a result with a positive value indicates an increase in properties, while a negative value means there has been a decrease.
